# Antenatal sildenafil administration to prevent pulmonary hypertension in congenital diaphragmatic hernia (SToP-PH): study protocol for a phase I/IIb placenta transfer and safety study

**DOI:** 10.1186/s13063-018-2897-8

**Published:** 2018-09-27

**Authors:** Francesca Maria Russo, Alexandra Benachi, Tim Van Mieghem, Jan De Hoon, Kristel Van Calsteren, Pieter Annaert, Jean-Marc Tréluyer, Karel Allegaert, Jan Deprest

**Affiliations:** 1 Department of Obstetrics and Gynecology, University Hospitals Leuven, Herestraat 49, 3000 Leuven, Belgium; 2 Cluster Woman and Child, Department of Development and Regeneration, Biomedical Sciences, KU Leuven, Leuven, Belgium; 3 Department of Obstetrics and Gynecology, Hôpital Antoine Béclère. AP-HP, Université Paris Sud, Clamart, France; 4 Centre Référence Maladie Rare: Hernie de Coupole Diaphragmatique, Paris, France; 5 Departments of Obstetrics and Gynaecology, Mount Sinai Hospital and University of Toronto, Toronto, ON Canada; 6 Center for Clinical Pharmacology, University Hospitals of Leuven, KU Leuven, Leuven, Belgium; 7 Drug Delivery and Disposition, Department of Pharmaceutical and Pharmacological Sciences, KU Leuven, Leuven, Belgium; 8 Pharmacologie et Recherche Clinique Paris Descartes Cochin Necker, Paris, France; 9 Institute of Women’s Health, Institute of Child Health, University College London, London, UK; 10 Department of Pediatrics, Division of Neonatology, Erasmus MC Sophia Children’s Hospital, Rotterdam, the Netherlands

**Keywords:** Sildenafil citrate, Congenital diaphragmatic hernia, Transplacental therapy, Placenta transfer study

## Abstract

**Background:**

Congenital diaphragmatic hernia is an orphan disease with high neonatal mortality and significant morbidity. An important cause for this is pulmonary hypertension, for which no effective postnatal therapy is available to date. An innovative strategy aiming at treating or preventing pulmonary hypertension more effectively is urgently needed. Prenatal sildenafil administration to expectant mothers prevented fetal and neonatal vascular changes leading to pulmonary hypertension in several animal models, and is, therefore, a promising approach. Before transferring this antenatal medical approach to the clinic, more information is needed on transplacental transfer and safety of sildenafil in humans.

**Methods:**

This is a randomized, investigator-blinded, double-armed, parallel-group, phase I/IIb study with as a primary objective to measure the in-vivo transplacental transfer of sildenafil in women in the second and early third trimester of pregnancy (sub-study 1; weeks: 20.0–32.6) and at term (sub-study 2; weeks: 36.6–40). Participants will be randomized to two different sildenafil doses: 25 or 75 mg. In sub-study 1, a single dose of the investigational product will be administered to women undergoing termination of pregnancy, and maternal and fetal blood samples will be collected for determination of sildenafil concentrations. In sub-study 2, sildenafil will be administered three times daily from 3 days before planned delivery until actual delivery, following which maternal and umbilical cord samples will be collected. Proxies of maternal and fetal tolerance as well as markers of fetal pulmonary vasodilation will also be measured.

**Discussion:**

This is the first study evaluating in-vivo transplacental passage of sildenafil in humans.

**Trial registration:**

EU Clinical Trials Register 2016–002619-17, validated on 12 August 2016.

Trial sponsor: UZ Leuven, Herestraat 49, 3000 Leuven.

**Electronic supplementary material:**

The online version of this article (10.1186/s13063-018-2897-8) contains supplementary material, which is available to authorized users.

## Background

Congenital diaphragmatic hernia (CDH) is a birth defect occurring in 1/2500 newborns and is characterized by a failure of the diaphragm to fully form in early pregnancy. This causes herniation of the abdominal organs into the thorax, which compromises lung development, eventually leading to pulmonary hypoplasia [1]. At birth, the clinical consequence is respiratory insufficiency and persistent pulmonary hypertension (PHT) which cannot simply be solved by surgically repairing the diaphragm. Thanks to advances in postnatal management, survival of neonates with CDH has increased to 70% [2–4]. Despite that, PHT remains a major determinant of mortality and morbidity, with only expert opinions to guide treatment [5, 6].

Today, more than 70% of CDH cases are diagnosed prenatally, following which additional testing is done to estimate the degree of fetal pulmonary hypoplasia and to provide a personalized prognosis [7–11]. The ability to identify a future non-survivor during pregnancy sparks the question for a prenatal intervention to reverse the natural course of the condition. Prenatal lung growth can be stimulated by minimally invasive tracheal occlusion (TO) [12]. The efficacy of this procedure currently undergoes evaluation in a global, open-label randomized clinical trial (https://totaltrial.eu/) [13]. Although TO improves lung size and may thus increase postnatal survival, it only partially improves PHT. Furthermore, TO remains a surgical intervention, with its inherent side effects and complications, such as prematurity, partially offsetting the effect of fetal therapy [14, 15].

As an alternative or complementary treatment to TO, we will investigate the use of transplacentally administered sildenafil. Sildenafil is a selective inhibitor of phosphodiesterase-type-5. It is primarily marketed for the treatment of erectile dysfunction, but also for treating PHT in adults [16]. It enhances nitric oxide (NO)-mediated vasodilation [17], promotes pulmonary angiogenesis and decreases pulmonary arterial remodeling [18, 19]. The drug is effective in children above 1 year of age who have PHT [20]. There is a growing interest in the use of sildenafil for the treatment of PHT of various etiologies in the newborn, including in CDH [21], particularly when other therapies, such as inhaled NO, fail. Such step-up strategy has been recently incorporated in the updated European “Euro-CDH Consortium” consensus neonatal management protocol for CDH infants [22].

Sildenafil has also already been used in pregnant women for maternal PHT or for pregnancy complications such as pre-eclampsia and intrauterine growth restriction [23, 24]. A systematic review summarizing clinical studies on sildenafil use in pregnancy has shown that maternal tolerance and perinatal outcomes are comparable with controls [25]. All this suggests a safe use of sildenafil from mid-gestation onwards.

Given the maternal and fetal safety profile of sildenafil, and given its established use in the postnatal treatment of PHT, the logical next step is to consider sildenafil use *prenatally*. Indeed, this is when the morphologic basis for PHT is being laid down, and, as such, prenatal treatment could potentially prevent PHT.

In two animal models of CDH, sildenafil has been shown to rescue the pulmonary vascular bed [26, 27]. Luong et al. were the first to demonstrate that maternal sildenafil administration reverses the morphologic pulmonary vascular changes associated to CDH in the nitrofen rat model [26]. Given our focus on making fetal interventions for CDH more effective and less invasive, we felt the need to move the concept of sildenafil further along the translational research track. We demonstrated the safety and efficacy of sildenafil in the rabbit [27]. Placebo-exposed CDH fetuses had an increased wall thickness in peripheral pulmonary vessels and significantly less fifth-generation vessels compared to controls. CDH fetuses treated with sildenafil had a medial thickness in peripheral pulmonary vessels in the normal range and a normal percentage of vessels of the fifth generation or higher. Sildenafil also reversed the mean terminal bronchiolar density in CDH fetuses to normal values and improved lung mechanics. Functionally, we could demonstrate reduced in-utero pulmonary vascular resistance by micro-ultrasound Doppler examination. No maternal or fetal adverse effects were observed. In summary, both in the rodent and the rabbit model, antenatal sildenafil administration rescues vascular branching and architecture and improves airway morphometry. These preclinical findings, together with the demonstration of the safety of sildenafil during pregnancy, pave the way for clinical translation.

One last critical step prior to clinical translation is that we need a better knowledge of sildenafil pharmacokinetics and transplacental transfer in vivo. This information is currently not available in the academic literature. This knowledge gap is explained by the fact that the product is currently used for maternal indications or to improve the flow in the uterus and placenta [24, 28, 29]. In these conditions fetal effects are less relevant, hence they were less studied and fetal drug levels were not measured. We have recently performed ex-vivo placental transfer studies [30]. We demonstrated that sildenafil crosses the placenta ex vivo at a rate that is independent from the initial maternal concentration and that there is sufficient placental transfer to reach therapeutic fetal drug levels at non-toxic maternal concentrations. These results need now to be validated in vivo.

## Methods and trial design

### Objectives

The main objective of the study is to evaluate the transfer of sildenafil through the second and third trimester human placenta in vivo.

Secondary objectives are:

- To evaluate maternal, fetal and neonatal tolerance
- To evaluate the effect of sildenafil on the fetal pulmonary circulation

### Trial design

This is a randomized, investigator-blinded, double-armed, parallel-group, phase I/IIb study consisting of two different sub-studies recruiting simultaneously:

- Second trimester placental transfer study (sub-study 1)
  The primary outcome of this sub-study is the fetal plasma sildenafil level after oral administration maternally. Fetal plasma will be obtained by fetal cardiocentesis or cordocentesis, required for administration of the drugs used for feticide at the time of pregnancy termination [31, 32]. Maternal blood sampling will be performed simultaneously via peripheral venous puncture.
  Secondary endpoints are:
  - Doppler ultrasound measures of pulmonary vasodilatation: change in the acceleration time/ejection time (AT/ET) ratio of the main fetal pulmonary artery and of the pulmonary artery branch closest to the ultrasound probe between baseline and the time of fetal blood sampling. Additional measures of blood flow resistance in the fetal main pulmonary artery and in the right and left pulmonary artery branch (Pulsatility Index, Resistance Index, peak systolic velocity, peak early diastolic reversed flow) will also be collected
  - Effect of sildenafil on the fetal systemic circulation: change in the Doppler waveform of the umbilical artery, between baseline and blood sampling
  - Simulated maternal and fetal pharmacokinetic profiles
  - Maternal tolerance, measured as change in blood pressure, heart rate and oxygen saturation compared to baseline, and incidence of side effects, including any kind of visual disturbance
- Third trimester placental transfer and safety study (sub-study 2)
  The primary endpoint is sildenafil transfer at term, expressed as a ratio of the neonatal over the maternal sildenafil plasma concentration. Neonatal venous blood samples will be collected from the umbilical cord at delivery. Maternal blood sampling will be performed via peripheral venous puncture.
  Secondary endpoints are:
  - Doppler ultrasound measures of pulmonary vasodilatation as described above
  - Effect of sildenafil on the fetal systemic circulation: change from baseline in the Doppler waveform of the umbilical artery, mean cerebral artery, ductus venosus, change in fetal heart rate monitoring pattern
  - Effect of sildenafil on the maternal placental circulation: change from baseline in the Doppler waveform of the uterine arteries
  - Simulated maternal and fetal pharmacokinetic profiles
  - Maternal tolerance measured as change in blood pressure, heart rate and oxygen saturation compared to baseline, and incidence of side effects, including any kind of visual disturbance
  - Neonatal tolerance, measured as trend in mean arterial blood pressure during the first 24 h of life, vasoactive inotropic score [33] at 24 h of life and early (< 24 h) lung function, expressed as Oxygenation Index and alveolar-arterial oxygen gradient

For both sub-studies, participants will be randomized to one of two parallel groups receiving different doses of sildenafil (sildenafil 25 or 75 mg). No placebo control is foreseen. The protocol follows the Standard Protocol Items: Recommendations for Interventional Trials (SPIRIT) recommendations for interventional trials (Additional file 1). 

### Study population

#### Second trimester placental transfer study (sub-study 1)

##### Inclusion criteria

- Volunteer with singleton pregnancy undergoing second trimester or early third trimester termination of pregnancy for fetal anomalies not interfering with normal lung development
- Maternal age > 18 and < 45 years (to allow consent and avoid age-related cardiac risks, respectively)

##### Exclusion criteria

- Multiple pregnancy
- Maternal diseases (such as hypertension, diabetes mellitus, infections), pregnancy complications (such as pre-eclampsia or growth restriction) or use of medication that could interfere with placental transfer of the study drug
- Contraindication to sildenafil therapy: renal or hepatic failure, maternal cardiac disease, hypotension (blood pressure < 90/60 mmHg), stroke, retinitis pigmentosa, sickle cell disease, bleeding disorders, active peptic ulcer, concomitant treatment with nitrates, previous serious adverse reaction or allergy to sildenafil, use of other vasodilator therapy, alpha-blockers, protease inhibitors, erythromycin or cimetidine
- History of psychiatric or psychological disease or concerns about psychological eligibility
- Previous loss of vision because of suspected non-arteritic, anterior, ischemic optic neuropathy (NAION)

#### Third trimester placenta transfer and safety study

##### Inclusion criteria

- Singleton pregnancy with isolated fetal CDH, left- or right-sided, with any degree of lung hypoplasia
- Gestational age ≥ 36 + 6 and ≤ 40 weeks
- Planned delivery at least 3 days after study initiation
- Maternal age ≥ 18 and ≤ 45 years (to allow consent and avoid age-related cardiac risks respectively)

##### Exclusion criteria

- Multiple pregnancy, chromosomal or major structural fetal abnormality
- Maternal conditions (such as hypertension, diabetes mellitus, infections), pregnancy complications (such as pre-eclampsia or growth restriction) or use of medication that could interfere with placental transfer of the study drug
- Any contraindication to sildenafil therapy as in sub-study 1
- Preterm premature rupture of membranes (pPROM)

### Interventions

In both sub-studies, sildenafil tablets will be administered orally to the mother as this administration route is the most practical and clinically most acceptable for sustained administration, especially in pregnant women. The half-life of sildenafil after oral administration is about 4 h [34]. Therefore, the most commonly used administration schemes for chronical conditions even in pregnancy consist in three administrations daily [20, 35–37].

Two sildenafil doses have been selected according to previously published literature and to our ex-vivo data on placental transfer [30], i.e., 25 and 75 mg. A dose of 25 mg three times daily has been proven safe in the treatment of pregnancies complicated by intrauterine growth retardation (IUGR) [24]. A dose of 75 mg three times daily is the maximum dose used in a trial on pregnant women affected by pre-eclampsia, without any fetal or maternal side effects being reported under that dose [23].

#### Second trimester placental transfer study (sub-study 1)

A single administration of sildenafil is planned before maternal and fetal blood sampling. Participants will be randomized to receive either a 25-mg or a 75-mg dose. For each dose, participating women will additionally be randomized to receive sildenafil either 2 h or 8 h before the termination. These time points have been selected because they represent the peak time and the trough time, respectively [34].

#### Third trimester placental transfer and safety study (sub-study 2)

Participants will be allocated to receive sildenafil tablets at a dose of 25 mg or 75 mg three times daily from 3 days prior to planned delivery until delivery.

**Fig. 1:**
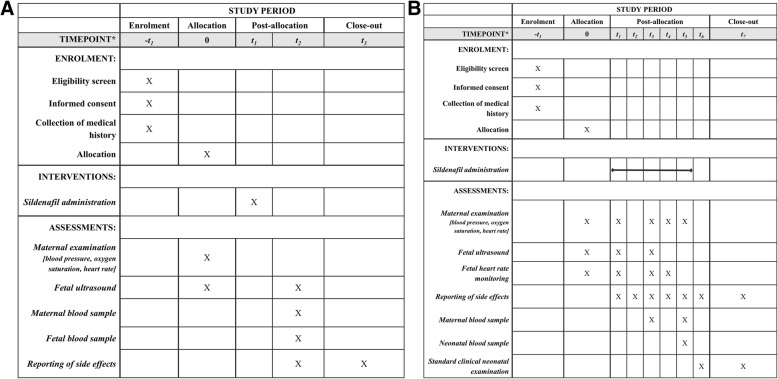
A; * − t_1_: screen visit; t0: allocation/baseline visit; t_1_: 2 or 8 h before termination of pregnancy; t_2_: at the time of feticide; t_3_: at patient’s discharge. B: * − t_1_: screen visit; t0: allocation/baseline visit; t_1_: 3 days before induction; t_2_: day 1 after first sildenafil administration; t_3_: 50 h after first sildenafil administration; t_4_: at beginning of labor; t_5_: at delivery; t_6_: day 1 after delivery; t_7_: at maternal and neonatal discharge

The Standard Protocol Items: Recommendations for Interventional Trials (SPIRIT) Diagram in Fig. 1 summarizes trial procedures for sub-studies 1 (Fig. 1A) and 2 (Fig. 1B) [38]. For both sub-studies, collection of routine clinical information from participants’ medical records to determine eligibility, informed consent and patient randomization will take place during the screening visit. A web-based randomization tool will be used in order to assign each patient to a study arm.

#### Second trimester placental transfer study (sub-study 1)

- Eligible patients will be randomly assigned to one of four different study groups: dose 1 (25 mg) with sampling at 2 h, dose 2 (75 mg) with sampling at 2 h, dose 1 with sampling at 8 h or dose 2 with sampling at 8 h
- Baseline visit: maternal blood pressure, heart rate and oxygen saturation will be measured and Doppler examination of the fetal pulmonary circulation and umbilical artery will be performed
- Day 0: at the time of pregnancy termination, the same procedures as in the baseline visit will be performed. At this time point, one maternal and one fetal blood sample will be collected for analysis of sildenafil concentration

#### Third trimester placental transfer and safety study (sub-study 2)

- Participants will be randomly assigned to one of two study groups: dose 1 (25 mg three times daily) and dose 2 (75 mg three times daily)
- Baseline visit: the study procedures will involve measurements of maternal blood pressure, heart rate and oxygen saturation; fetal ultrasound for assessment of fetal wellbeing and Doppler examination of the fetal main pulmonary artery and contralateral pulmonary artery branch, mean cerebral artery, umbilical artery, ductus venosus; Doppler examination of the uterine arteries; fetal heart rate monitoring (cardiotocography)
- During sildenafil administration (from day 0 until occurrence of labor, specifically at 2 and 50 h after the first administration) the study procedures will be the same as in the baseline visit. Treatment compliance and any side effect will also be reported. One maternal blood sample will be collected on day 2 (50 h after the first administration) for determination of maternal plasma sildenafil concentration
- During labor and at delivery fetal heart rate, maternal blood pressure, heart rate and oxygen saturation, and eventual side effects will be recorded as part of the clinical protocol. At the moment of delivery, one maternal and one umbilical cord blood sample will be collected for determination of sildenafil concentration
- At the neonatal follow-up visit (day 1 after birth), mean arterial blood pressure, vasoactive inotropic score [33] and early (< 24 h) lung function, expressed as Oxygenation Index and alveolar-arterial oxygen gradient will be recorded. Adverse effects, if any, will be recorded

### Sample size calculation

This is a phase II, dose-finding study and there is no information in the literature about transplacental sildenafil transfer in vivo. A formal power calculation has, therefore, not been performed [39, 40]. The number of cases is driven by the practices commonly used to document pharmacokinetics in a rather homogeneous population with simultaneous collection of maternal and fetal effects.

#### Second trimester placental transfer study (sub-study 1)

We assume that a sample size of six participants per dose for each time point (total = 24 women) is an ethical balance between the research question and the exposure of healthy volunteers to the drug. In addition we will perform an interim analysis after having obtained all data in 16 women (four per group) to potentially conclude the study prematurely in case of a low variability (< 20% variability in fetal sildenafil concentrations) within the groups. To ensure a balance in sample size across groups over time, a block size randomization of 8 with equal numbers for each group is chosen [41, 42].

We expect a 2% incidence of maternal conditions representing an exclusion criteria for the study. Furthermore, since this is a study on pregnant women without medical conditions, we expect a high rejection rate (~ 50%). Since this is a short-term study, we do not expect any drop-out after allocation. Therefore, the plan is to assess a total of 49 women for eligibility for recruitment of up to 24 women.

#### Third trimester placental transfer and safety study (sub-study 2)

To obtain reliable pharmacokinetic data, we plan to include six patients for each tested dose (total = 12 patients). For the reasons given above in sub-study 1, block randomization (block size = 4) will be used.

We expect a 50% incidence of maternal or fetal (i.e., not isolated cases, fetuses having had fetal therapy) conditions representing an exclusion criterion for the study and a 50% rejection rate. Speculating that the drop-out rate after inclusion will be less than 10%, we will need to assess for eligibility a total of 52 women with ongoing pregnancies and CDH at term.

The Data Safety and Monitoring Committee will evaluate drop-out rate ad interim, and is authorized to adjust sample size, if appropriate.

### Statistical analysis plan

For all pharmacokinetic, demographic, safety and efficacy outputs, data will be summarized by sub-study and dose group. For continuous variables, summary statistics will include the number of observations, the arithmetic mean, median, arithmetic standard deviation, minimum and maximum. For categorical variables, frequency counts and percentages will be used.

The measured sildenafil concentrations will be used to develop a physiologically based pharmacokinetic (PBPK) model allowing prediction of the maternal dose that will result in therapeutic concentrations in the fetus [43]. The effect of covariates (gestational age, maternal height and weight) will also be assessed.

For efficacy and tolerability endpoints, baseline is defined as the last value for each assessment prior to the subject’s first dose. Each individual change from baseline will be calculated by subtracting the individual subject’s baseline value from the value at the time point. The individual subject’s change from baseline values will be used to calculate the mean change from baseline. Missing values will not be imputed.

Adverse events will be summarized for each of the sub-studies and of the dose groups. Summaries will include the number of patients who have at least one occurrence of the adverse event. In addition, the total number of adverse events with a first reporting of the event while on treatment will be displayed. The analysis will be for descriptive purpose only and no inferential testing was performed.

## Discussion

The mortality from CDH-related PHT unresponsive to medical therapy remains around 60% [44], and infants with PHT require prolonged mechanical ventilation, need extracorporeal life support, or progress into right-sided heart failure [44, 45]. PHT also adversely affects the quality of life in CDH survivors [46]. CDH, certainly when complicated by PHT, also is a serious socio-economic burden. The postnatal management costs of a CDH patient is estimated to exceed US$200,000 [47], making it the costliest non-cardiac birth defect [48]. When the baby also has PHT, costs are significantly higher. For instance, when extra-corporeal membrane oxygenation is used (which is more likely in cases of PHT), in-hospital costs rise fourfold. To this, the burden of chronic PHT must be added [49]. Chronic PHT is associated with an increased risk of death, cardiac and pulmonary complications and re-hospitalization. It requires chronic medical treatment, an intense clinical follow-up often with additional testing and investigations.

Therefore, effective treatment or prevention of PHT is urgently needed. SToP-PH introduces the concept of a medical approach that acts already during fetal life hence preventing the ill-development of pulmonary vasculature. The herein described phase I studies are required to properly design a randomized controlled trial on maternal sildenafil administration for the prevention of PHT in CDH. If proven effective, oral administration of a safe drug will also allow for a wider implementation than fetal surgery, including in less privileged countries. Furthermore, sildenafil is generically available. Therefore, this would be the first affordable therapy for a fetal surgical condition ever.

The European Commission has supported the establishment of virtual thematic networks across Europe (European Reference Networks), aimed at sharing knowledge, exchanging information and facilitating collaborations between specialized centers. “ERNICA” is the European reference network on rare diseases of the foregut grouping skills from 20 centers in 11 countries. It covers malformations of the digestive system, the diaphragm (including CDH) and the abdominal wall. Such network will make a randomized clinical trial on CDH fetuses possible, despite the rarity of the condition. We also have already obtained Orphan Designation for this indication by the European Medical Agency (http://www.ema.europa.eu/docs/en_GB/document_library/Orphan_designation/2017/07/WC500231579.pdf). The present phase I/IIb trial should allow to determine the effective maternal dosage to be used in the subsequent phase III efficacy study. Moreover, it will investigate and provide first data on maternal and fetal tolerance and on the effect of the drug on the fetal pulmonary circulation. This knowledge will provide safety information also for other maternal and placental diseases, for which sildenafil is currently being explored.

## Trial status

### Recruiting

Approved by the Belgian National Authorities: 20 July 2017.

Date of local Ethics Committee approval (Leuven): 22 September 2017 (protocol version 5, dated 28 July 2017).

Recruitment starting date: 1 February 2018.

## Additional file

Additional file 1: **Standard Protocol Items: Recommendations for Interventional Trials** (SPIRIT) 2013 Checklist: recommended items to address in a clinical trial protocol and related documents. (DOC 124 kb)

## Abbreviations

AT/ET: Acceleration time/ejection time ratio
CDH: Congenital diaphragmatic hernia
NO: Nitric oxide
PHT: Pulmonary hypertension
TO: Tracheal occlusion
